# Endovascular aneurysm closure during out of office hours is not related to complications or outcome

**DOI:** 10.1007/s00234-019-02355-1

**Published:** 2020-02-07

**Authors:** Jasper H. van Lieshout, Dagmar Verbaan, Igor Fischer, Hendrik-Jan Mijderwijk, René van den Berg, W. Peter Vandertop, Catharina J.M. Klijn, Hans J. Steiger, Joost de Vries, Ronald H.M.A. Bartels, Kerim Beseoglu, Hieronymus D. Boogaarts

**Affiliations:** 1 Department of Neurosurgery, Medical Faculty, Heinrich-Heine-University, Moorenstraße 5, 40225 Düsseldorf, Germany; 2 Neurosurgical Center Amsterdam, Amsterdam University Medical Centers, Amsterdam, the Netherlands; 3 Divisions of Informatics and Statistics, Department of Neurosurgery, University Clinic Düsseldorf, Düsseldorf, Germany; 4 Departments of Radiology and Nuclear Medicine, Amsterdam University Medical Centers, Amsterdam, the Netherlands; 5 Department of Neurology and Donders Institute for Brain, Cognition and Behaviour, Radboud University Medical Center, Nijmegen, the Netherlands; 6 Department of Neurosurgery, Radboud University Medical Center, Nijmegen, the Netherlands

**Keywords:** Aneurysmal subarachnoid hemorrhage, Cohort study, Risk factors in epidemiology, Outcome research, Patient safety

## Abstract

**Purpose:**

A possible disadvantage of endovascular occlusion outside work hours is that complex procedures might expose patients to additional risk when performed in a suboptimal setting. In this prospective cohort study, we evaluated whether treatment during out of office hours is a risk factor for per-procedural complications and clinical outcome.

**Methods:**

We included 471 endovascular-treated, consecutive aneurysmal subarachnoid hemorrhage patients (56.6 ± 13.1, 69% female), from two prospective observational databases which were retrospectively analyzed. Primary outcome was the occurrence of per-procedural complications. Secondary outcomes were good clinical outcome (modified ranking scale ≤ 2) and death at 6-month follow-up. We determined odds ratios (OR) with 95% confidence intervals (CI) by ordered polytomous logistic regression analysis and adjusted odds ratios (aOR) for age, World Federation of Neurosurgical Societies grade, and time to treatment.

**Results:**

Most patients were treated during office hours (363/471; 77.1%). Treatment during out of office hours did not result in an increased risk of per-procedural complications (OR 0.85 (95% CI 0.53–1.37; *p* = 0.51). Patients treated during out of office hours displayed similar odds of good clinical outcome and death after 6 months (OR 1.14, 95% CI 0.68–1.97 and 1.16 95% CI 0.56–2.29, respectively) compared to patients treated during office hours.

**Conclusion:**

In our study, endovascular coil embolization during out of office hours did not expose patients to an increased risk of procedural complications or affect functional outcome after 6 months.

## Introduction

Early (≤ 72 h) aneurysm closure after intracranial aneurysm rupture is critical to prevent aneurysm rebleeding, and its importance is reflected in current guidelines [1, 2]. Some studies have found evidence for a benefit of ultra-early (≤ 24 h) or even emergency (≤ 6 h) treatment of ruptured aneurysms to reduce aneurysmal rebleeding rates and to improve clinical outcome [3, 4]. The potential benefit of emergency or ultra-early aneurysm treatment has been suggested to be more pronounced for endovascular coiling than for surgical clipping [4].

However, inconsistent results between studies lead to a lack of evidence on whether earlier endovascular treatment actually improves outcome in patients with aneurysmal subarachnoid hemorrhage (aSAH) [5]. As a result, timing of aneurysm closure remains controversial. In some centers, procedures are not performed during out of office hours, because of less optimal logistics and expertise, whereas others favor immediate closure of the aneurysm irrespective of the time of presentation. Van Lieshout et al. recently showed a higher per-procedural re-rupture and a higher probability of poor outcome after emergency coiling (≤ 6 h) in comparison with coiling after 6 h [6]. A possible explanation for this higher risk may be that patients treated on an emergency basis are more likely to be treated during out of office hours. We hypothesized that patients treated during out of office hours are more likely to experience per-procedural complications and are at higher odds of poor outcome compared to those treated during office hours.

## Methods

### Study population and inclusion

We included 471 consecutive patients with aSAH from two prospective observational databases, at the neurovascular centers from the Radboud University Medical Center (Radboudumc, *n*=233), Nijmegen, and the Amsterdam University Medical Centers (location AMC, *n*=238), Amsterdam, the Netherlands, between January 2012 and January 2016. Patients from the ultra-early tranexamic acid after subarachnoid hemorrhage (ULTRA) study were not included [7]. Only patients with a ruptured intracranial aneurysm who were treated by endovascular coil embolization were included. The institutional review boards confirmed that the Medical Research Involving Human Subjects Act (WMO) does not apply and that official approval for this study is not required (reference number: 3449 Radboudumc and 17286 for the AMC).

### Data collection and organization of the register

We obtained all data from the Quality Registry Neurosurgery (QRNS) of the Dutch national neurosurgical society (NVvN), a physician-driven prospective national outcome register for aSAH. We obtained the following data: patient characteristics (age, sex), clinical and radiological (Fisher grade, aneurysm size) characteristics, time of ictus and treatment, treatment modality, per-procedural complications, and clinical outcome. Reporting of this study was according to the Strengthening the Reporting of Observational Studies in Epidemiology (STROBE) guidelines for observational studies (supplementary material) [8].

### Treatment protocol

All admitted aSAH patients undergo a standardized treatment protocol, as previously proposed elsewhere [1, 2]. Both centers aim for aneurysm closure within 8 and at the latest 24 h after ictus, regardless of aneurysm complexity. For patients in poor clinical condition, World Federation of Neurosurgical Societies (WFNS) grade 5 and timing of treatment were variable and could have been postponed in individual cases.

At the Radboudumc, hybrid vascular neurosurgeons evaluate radiological imaging and make treatment decisions, whereas at the AMC interventional neuroradiologists and vascular neurosurgeons do so in consensus. Both centers provide a 24/7 consultant cover of services. The neuro-interventional specialists who performed the endovascular procedures all had at least 5 years of experience in endovascular treatment of aneurysms and aSAH.

### Definitions and outcome parameters

We defined the time to treatment as the time interval between ictus of hemorrhage and start of endovascular aneurysm closure. The primary outcome of this study was the occurrence of per-procedural complications: aneurysm perforation during the procedure, ischemia attributed to endovascular treatment, thrombus formation, and arterial dissection. The occurrence of any per-procedural complications was scored as a binary event, yes (1) or no (0). We defined office hours from 8:00 a.m. to 5:59 p.m. and out of office hours from 6:00 p.m. to 7:59 a.m.

The secondary outcomes of the study were death and clinical outcome at 6 months after treatment, measured by the modified Rankin scale (mRS) score and determined by a specialized nurse who had not been involved in the patients’ treatment. Good clinical outcome was defined as mRS ≤ 2 [9]. The mRS was measured with a standardized, validated structured interview.

### Data analysis

Categorical data are presented as numbers (percentages) and continuous variables as mean ± standard deviation (SD) or median with interquartile range (IQR) depending on the distribution.

We tested categorical data with the Pearson chi-square test and continuous data with the Student’s *t* test for independent observations or the Mann-Whitney test (Wilcoxon signed-rank test) as appropriate. We used binary logistic regression analysis to evaluate if time to treatment (during office hours or out of office hours) was associated with per-procedural complications (primary outcome). Ordered polytomous logistic regression analysis (link function: logit) was used to evaluate if time to treatment (during office hours or out of office hours) was associated with clinical outcome after 6 months (secondary outcome). Both analyses were adjusted for age, WNFS grade, and time between ictus and treatment, based on subject matter knowledge. *p* values (two-tailed) and confidence intervals were estimated for all parameters. Patients with missing data or those lost to follow-up were excluded from further analysis for the corresponding analysis.

The type I error was set at 0.05 and the tests were two-tailed. For statistical analysis, we used SPSS software version 25.0 (SPSS Institute, Chicago, IL, USA) and the R statistical computing package, R version 3.4.2 (R Foundation for statistical computing, Vienna, Austria; URL: https://www.R-project.org/).

## Results

### Demographics

We included 471 patients (mean age 56.6; SD ± 13.1; 69% female). Characteristics of the study population are listed in Table 1. Univariate analysis revealed no differences between the two groups except for time to treatment. Patients treated during out of office hours were treated earlier after ictus compared to those treated during office hours (Table 1). The dataset was incomplete for 19/471 patients (4%).

**Table 1:** Patient characteristics at baseline distinguished by time of treatment

	All patients (*n* = 471)	Office hours (*n* = 363)	Out of office hours (*n* = 107)	*p* value
Age, mean ± SD	56.6 ± 13.1	56.9 ± 12.8	55.7 ± 14.1	0.181 ^Ɨ^
Female, *n* (%)	326 (69.2)	245 (67.5)	81 (75.7)	0.107^*^
WFNS grade, *n* (%)	462	358	104	0.163^*^
I	203 (43.9)	154 (43)	49 (47.1)	
II	100 (21.6)	82 (22.9)	18 (17.3)	
III	22 (4.8)	13 (3.6)	9 (8.6)	
IV	58 (12.6)	44 (12.3)	14 (13.5)	
V	79 (17.1)	65 (18.2)	14 (13.5)	
Fisher grade, *n* (%)	469	362	107	0.672^*^
I	14 (3)	11 (3)	3 (2.8)	
II	44 (9.4)	31 (8.6)	13 (12.1)	
III	103 (22)	79 (21.8)	24 (22.4)	
IV	308 (65.6)	241 (66.6)	67 (62.6)	
Time to treatment, median [IQR]	19 [10–42]	20 [11.5–46.3]	12 [7–24]	0.001^¥^

ƗStudent T-test; *Pearson chi-square test; ¥Mann-Whitney U-test

### Time of treatment

**Figure 1:**
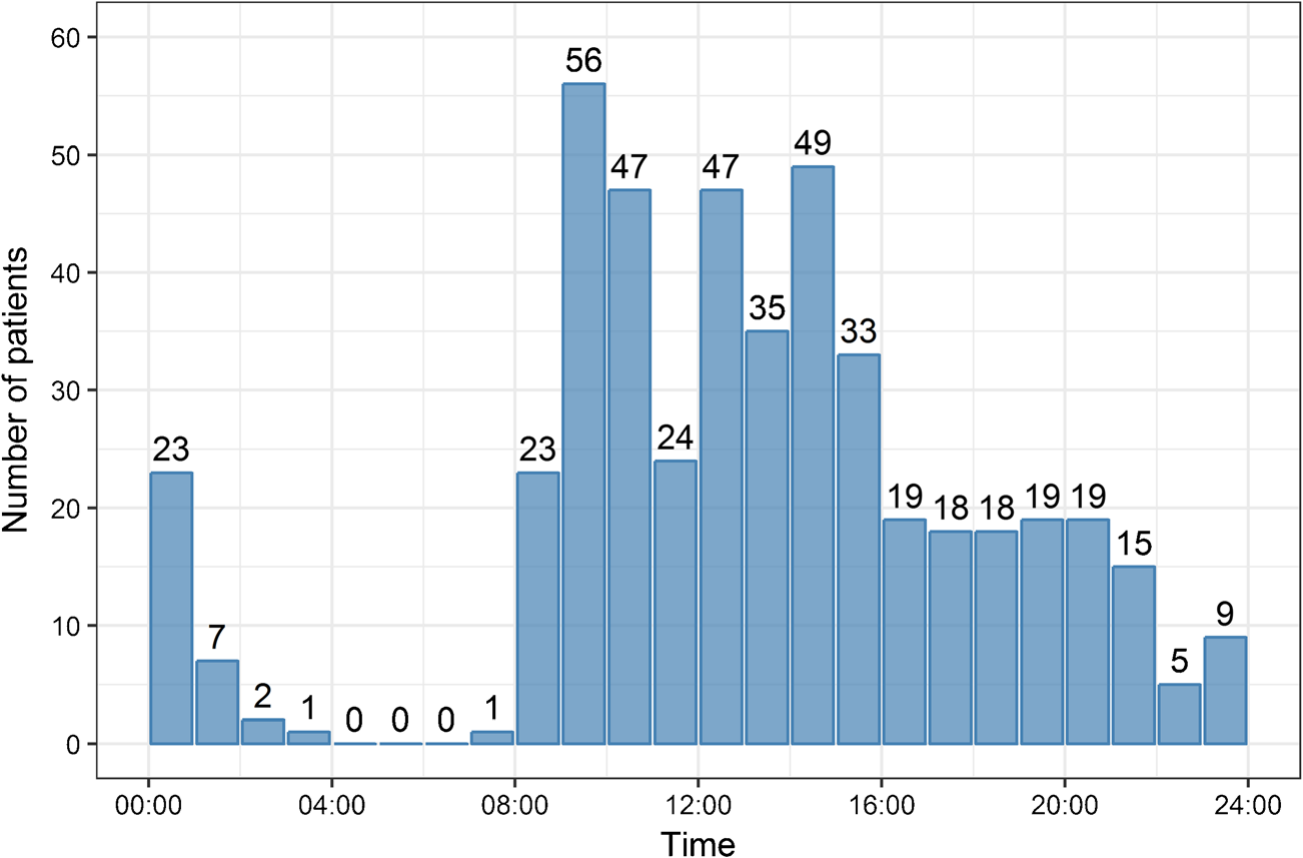
This illustrates the distribution of the aneurysms according to the starting time of the endovascular treatment during the day

We treated more patients during office hours (363/471; 77.1%) than during out of office hours (107/471; 22.7%, Table 1). In most patients, aneurysm closure was performed between 8:00 and 16:00 as expected. None of the endovascular treatments was started between 4:00 and 7:00, and a minority was started between midnight and 2:00 (Fig. 1). Around a third of the patients with an ictus during office hours were eventually treated during out of office hours (74/253; 29.2%) compared to 15.3% of the patients with an ictus during out of office hours (33/215).

### Per-procedural complications and clinical outcome during out of office hours

Treatment during out of office hours did not result in an increased risk of per-procedural complications (Table 2). Adjustment for age, WFNS grade, and time to treatment did not change the results (Table 2). Information on clinical outcome 6 months after treatment was available for 96 (90.5%) patients treated during out of office hours and for 327 (89.8%) patients in the office hour group. Patients treated during out of office hours were at similar odds of good clinical outcome and death at 6 months compared to patients treated during office hours (Table 3). There seems to be a bias for the availability of a mRS score after 6 months based on the WFNS grade (Table 4). However, the ORs remained similar after adjustment for age, WFNS grade, and time to treatment (Table 3). The proportional odds assumption was met.

**Table 2:** Complications of endovascular treatment distinguished by time of treatment

	All patients	Office hours	Out of office hours	OR (95% CI)	aOR (95% CI)
Overall (*n*, %)	55/469 (11.8)	41/364 (11.2)	14/106 (12.2)	0.88 (0.53–1.43)	0.85 (0.53–1.43)
Perforation (*n*, %)	12/469 (2.6)	9/364 (2.5)	3/105 (2.9)	0.99 (0.26–3.71)	0.94 (0.25–3.58)
Ischemia (*n*, %)	29/468 (6.2)	25/363 (6.9)	4/105 (3.8)	0.60 (0.22–1.60)	0.53 (0.18–1.6)
Thrombus (*n*, %)	48/468 (10.3)	36/363 (9.9)	12/105 (11.4)	1.10 (0.56–2.17)	1.16 (0.58–2.34)
Dissection (*n*, %)	1/459 (0.2)	1/355 (0.3)	0/104 (0)	0 (na)	0 (na)

Logistic regression analysis; na = not applicable

**Table 3:** Functional outcome after 6 months

	All patients (*n* = 424)	Office hours (*n* = 327)	Out of office hours (*n* = 97)	OR (95% CI)	aOR (95% CI)
mRS, *n* (%)					
mRS ≤ 2	290 (68.4)	222 (67.9)	68 (70.1)	1.11 (0.65–1.90)	1.14 (0.68–1.97)
mRS = 6	56 (13.2)	42 (12.8)	14 (14.6)	1.09 (0.63–1.85)	1.16 (0.56–2.29)

Logistic regression analysis

**Table 4:** Patient characteristics at baseline distinguished availability of mRS score after 6 months

	All patients (*n* = 471)	mRS available (*n* = 424)	no mRS available (*n* = 47)	*p* value
Age, mean ± SD	56.6 ± 13.1	56.5 ± 12.9	57.3 ± 14.8	0.415 ^Ɨ^
Female, *n* (%)	327 (69.2)	298 (70.2)	29 (61.7)	0.244^*^
WFNS grade, *n* (%)	463	419	44	0.046^*^
I	204 (43.9)	179 (42.2)	25 (56.8)	
II	100 (21.6)	95 (22.4)	5 (11.4)	
III	22 (4.8)	22 (5.2)	0 (0)	
IV	58 (12.6)	49 (11.6)	9 (20.5)	
V	79 (17.1)	74 (17.5)	5 (11.4)	
Fisher grade, n (%)	469	422	47	0.533^*^
I	14 (3)	14 (3.3)	0 (0)	
II	44 (9.4)	41 (9.7)	3 (6.4)	
III	103 (22)	90 (21.2)	13 (27.7)	
IV	308 (65.6)	241 (56.8)	31 (66)	
Time to treatment, median [IQR]	19 [10–42]	18.5 [9.5–41]	24 [12.5–52]	0.074^¥^

ƗStudent T-test; *Pearson chi-square test; ¥Mann-Whitney U-test

## Discussion

In this study, endovascular coil embolization during out of office hours was not associated with an increased risk of treatment-related complications, poor functional outcome, or death after 6 months.

Healthcare professionals who treat patients with ischemic stroke are often the same who treat patients with aSAH. As a result, resources for the diagnosis and treatment of aSAH are available round-the-clock in many neurovascular centers. Nevertheless, a survey among German hospitals involved in treatment of aSAH showed that the majority of hospitals (98%) does not provide immediate aneurysm closure at night, be it by either endovascular or neurosurgical treatment [10]. Moreover, most hospitals do not start treatment later than 20:00. Both human and situational factors may cause decreased quality of care during nighttime, and indeed task performance on manual monitoring tasks has been found worse at night [11, 12]. It is therefore surmised that aneurysm repair during out of office hours increases the risk of treatment-related complications, based upon translation of results from nightly treatment of other diseases, although so far this has not been studied explicitly for endovascular closure of ruptured cerebral aneurysms [10, 13, 14].

Our results stand in contrast with findings from the before mentioned reports but recapitulates the results of a previous study, which show that nighttime surgery is not independently associated with higher intraoperative risk of complications [15].

Strengths of our study include the prospective data collection representing a homogenous national healthcare setting and a high follow-up rate. However, there are several limitations that need to be addressed. Since very few patients were treated between midnight and 8:00, it is challenging to draw conclusions on the risks of treatment during those early hours. Since we started treatment between 1:00 and 8:00 a.m. in just 11 patients, our results seem valid for a treatment onset time up to 1 a.m. Our data suggest that not the clinical status but the hour of day determines delay of endovascular aneurysm closure. As a result, none of the endovascular treatments started between 4:00 and 7:00, and only few treatments started between midnight and 4:00. Despite the collaboration between two referral centers, the number of patients with procedural complications was small, and not all potential relevant factors were collected in the prospective database, such as blood pressure on admission and during the procedure. Due to the disbalance of the number of patients between the groups, our study may be underpowered and limited by selection bias. Moreover, possible imprecision in determining time of ictus could have influenced the registered time to treatment and might therefore have altered our findings. Finally, this study was observational, and not a randomized comparison of patients presenting during out of office hours treated immediately or delayed, so firm conclusions cannot be drawn on the optimal timing of treatment.

Emergency aneurysm closure provides a theoretical benefit by eliminating the early risk of rebleeding. However it is questioned if in emergency aneurysm occlusion, the theoretical benefit of early repair might be offset by an increased risk of per-procedural complication of re-rupture [6]. The overall benefit of early endovascular aneurysm closure on outcomes is unclear, but aneurysm obliteration during out of office hours does not seem to have a negative impact on outcome and is therefore not an important argument against immediate treatment [5]. Due to the marked increased burden on resources, routine aneurysm closure during out of office hours would need a clear justification. Future efforts should focus on establishing whether emergency aneurysm closure improves patient outcome. If the beneficial effect of emergency aneurysm occlusion by reducing spontaneous rebleeding rates outweighs the higher rate of procedural aneurysm re-rupture, aneurysm closure after aSAH should be regarded as a neurological emergency similar to ischemic stroke [6].

## Conclusion

In this study, endovascular coil embolization during out of office hours was not associated with an increased risk of treatment-related complications, poor functional outcome, or death after 6 months. Few patients were treated at night, but we could not confirm the perceived notion that endovascular aneurysm treatment during out of office hours is associated with a higher risk of procedural complications.
